# Anticancer bioactivity of zerumbone on pediatric rhabdomyosarcoma cells

**DOI:** 10.1007/s00432-022-04237-1

**Published:** 2022-08-05

**Authors:** Cristian Urla, Matias Julian Stagno, Jörg Fuchs, Steven W. Warmann, Evi Schmid

**Affiliations:** Department of Pediatric Surgery and Pediatric Urology, University Children’s Hospital of Tuebingen, Hoppe-Seyler-Str. 3, 72076 Tuebingen, Germany

**Keywords:** Zerumbone, Pediatric, Rhabdomyosarcoma, Cell proliferation, Apoptosis

## Abstract

**Purpose:**

Natural products are generally regarded as safe and have been shown to mediate anticancer activities against a variety of cell types. Zerumbone is a natural cyclic sesquiterpene derived from the rhizome of Zingiber zerumbet, which has attracted extensive attention in the recent decade for anticancer activities. The present study investigates the in vitro effect of zerumbone on rhabdomyosarcoma cells.

**Methods:**

Two rhabdomyosarcoma cell lines (RD and RH30) were used as the model system. The growth inhibition of zerumbone was measured by MTT-assay, apoptosis via flow cytometry, gene expression by real-time PCR, the migration by transwell assay, and intracellular signaling by Western blotting.

**Results:**

Zerumbone shows anticancer effects on RD and RH30 cells in a dose-dependent manner via cell growth inhibition and induction of apoptosis. Exposure of RD and RH30 cells on zerumbone also resulted in a decrease of migration and downregulation of the hedgehog pathway.

**Conclusions:**

Taken together, our study provided the first evidence that zerumbone imparted strong inhibitory and apoptotic effects on pediatric rhabdomyosarcoma cell lines and merit further investigation as a promising candidate for the anticancer therapy.

## Introduction

Rhabdomyosarcoma (RMS) is the most common soft tissue sarcoma of children and adolescents, accounting for 5% of all malignant tumors in patients less than 15 years old (Yang et al. 2014). Most RMS tumors originate in the head and neck region, urogenital tract (bladder, prostate) and extremities (Loeb et al. 2008). Based on histology, there are two major variants of RMS—embryonal (RME) and alveolar (RMA) (Marshall and Grosveld 2012). RMA is more aggressive, has frequently worse outcome and is characterized by the presence of specific genetic alterations such as the chromosomal translocation: t(2;13)(q35;q14), seen in 55% of cases and t(1;13)(p36;q14), seen in 22% of cases. In contrast, no recurrent translocations have been identified in RME (McDowell 2003; Sorensen et al. 2002).

The treatment of the children suffering from RMS is multimodal and consists in chemotherapy and local tumor control with surgery and/or radiotherapy (Dantonello et al. 2009; Hayes-Jordan and Andrassy 2009). Multimodal therapy has led to an impressive improvement of outcomes for the vast majority of these children over the past three decades. However, little if any progress has been made in the treatment of patients with advanced stage tumors as many of these children will relapse after treatment (Dasgupta and Rodeberg 2012). Therefore, the development of novel therapeutic strategies for these patients is of eminent importance and future clinical trials will focus on the molecular biology driving RMS tumor behavior (Dasgupta and Rodeberg 2012).

In general, cancerous phenotypes result from the dysregulation of more than 500 genes at multiple steps in cell signaling pathway (Gupta et al. 2010; Vogelstein and Kinzler 2004). This indicates that inhibition of a single gene product or cell signaling pathway is unlikely to prevent or treat cancer. Therefore, the current paradigm for cancer treatment is either to combine several monotargeted drugs or to develop drugs that modulate multiple targets (Gupta et al. 2010).

During the past decade, the plant-derived dietary agents have drawn attention of researchers due to their multi-targeted potential (Gupta et al. 2010). Some of these compounds are currently in clinical trials, but others have already been approved for human use (Gupta et al. 2010; Amin et al. 2009).

Zerumbone is an extract from *Zingiber zerumbeta*, which exhibits anti-tumor and anti-inflammatory properties (Ma et al. 2018). Increased evidence indicates that zerumbone has chemoprotective and chemotherapeutic effects in various cancers, including liver (Sakinah et al. 2007), renal (Sun et al. 2013), cervix (Abdul et al. 2009), and colon cancer (Yodkeeree et al. 2009).

To our knowledge, the influence of zerumbone on pediatric RMS cells remains unknown. Therefore, the aim of the present study was to investigate whether zerumbone has an anti-tumor effect on RMS cells and to determine the mechanisms behind it.

## Materials and methods

### Cell lines and culture conditions

The RME cell line RD (ATCC, Manassas, VA, USA), the RMA cell line RH30 (DSMZ, Braunschweig, Germany), and the human skeletal muscle cells (SkMC; Sigma Aldrich, Taufkirchen, Germany) were routinely cultured in DMEM medium (Biochrom, Berlin, Germany) supplemented with 10% fetal bovine serum (Biochrom, Berlin, Germany), 1% penicillin/streptomycin (Biochrom, Berlin, Germany) and 1% l-glutamine (Biochrom, Berlin, Germany) in a humidified atmosphere containing 5% CO_2_ at 37 °C. Cell identity was proven by SLR analysis of the DNA profile using PowerPlex 16 (Promega, Mannheim, Germany). All cells were tested to be mycoplasma negative.

### Cell viability assay

Cells were seeded in 96-well plates at 8 × 103 cells per well in a triplicate. After overnight adherence of the cells and 72 h treatment with zerumbone, cell viability was determined by means of a colorimetric MTT-assay measuring the reduction of tetrazolium salts to formazan derivatives by functional mitochondria. Lysis buffer (DMSO, SDS, acid) was added to solubilize the blue MTT-formazan product. The assay was performed as originally described by Mosmann et al. (1983). Absorbance was measured at 570 nm. IC50 values were determined using GraphPad Prism version 8.0 (GraphPad Software, Inc.).

### Spheroid formation capacity

Multicellular tumor spheroids represent a complex model that imitates the in vivo situation. Due to their spherical structure, different properties result for different layers. The outer layer with the highest nutrient and oxygen supply proliferates, the middle layer consists of cells in the G0 phase and close to the center the cells necrotize due to the lack of nutrient and oxygen. This structure imitates micrometastases and in-vitro insights into expression behavior and metabolism can already be gained (Mueller-Klieser 1987; Sutherland and Durand 1984; Sutherland et al. 1971).

2 × 105 RH30 and RD cells were seeded in 100 µl previously filtered (Easystrainer 40 µM, Greiner Bio-One, Frickenhausen, Germany) cell culture medium onto a non-binding and U-bottom microplate (Thermo Fisher Scientific, Karlsruhe, Germany). Centrifugation (200 rpm, 5 min at RT) allowed the cells to form spherical aggregates. After incubation for 72 h, the tumor spheroids were treated with zerumbone for 72 h. Finally, the spheroids were incubated with 25 µl methylene blue (Biochrom, Berlin, Germany) per well for 24 h and measured on the multiplate reader (PerkinElmer, Waltham, USA) with the wavelengths 486 nm and 535 nm (fluorescein).

### Transwell migration assay

To verify if zerumbone has an influence on RMS cell migration a transwell migration assay was performed. For this purpose, 2.5 × 10^4^ to 5 × 10^4^ cells were plated in the top chamber with a non-coated membrane (24-well insert; pore size, 8 μm; BD Biosciences) (Schmid et al. 2012). Cells were plated in medium (Dulbecco’s Modified Eagle Medium) without serum, and medium supplemented with serum was used as a chemoattractant in the lower chamber. The cells were incubated with and without zerumbone for 24 h (RH30) or 48 h (RDs) in a humidified atmosphere at 37 °C and 5% CO_2_. Cells that did not migrate through the pores were removed by a cotton swab and washed with PBS. The transwells were moved to 4% paraformaldehyde (PFA) and incubated for 15 min at room temperature. Membranes were removed by scalpel, placed on slides and stained with Giemsa. The migrated cells bound on the lower surface to the membrane were then counted at 3 different regions using Axio Vision Release 4.8 software (Carl Zeiss Vision, Oberkochen, Germany).

### Colony-forming assay

RD and RH30 cells were plated in 6-well plates at 500 cells per well in 2 ml of media. After attachment cells were treated with zerumbone for 72 h in a humidified atmosphere of 37 °C and 5% CO_2_. After 72 h the cells were washed with PBS and fresh Media was added. The colonies have grown 7–10 days and afterward were fixed in 80% methanol, stained with 0.2% crystal violet and counted.

### Measurement of oxygen-reactive species (ROS) production

For FACS-based ROS measurements RD and RH30 cells were seeded in 6-well plates at a density of 1 × 10^5^ cells/well. Following attachment, the cells were treated with 25 and 50 µM zerumbone for 24 h. ROS measurement was monitored using DCHF-DA, which was added to the cells after treatment for 30 min at 37 °C. To exclude nonviable from viable cells, the attached cells were collected, washed twice with DMEM, treated with 5 µl 7-AAD (eBioscience, San Diego, USA) and incubated at room temperature in the dark for 15 min. Finally, 100 µl DMEM was added and ROS production was analyzed on a BD FACS CANTO II flow cytometer and evaluated with BD FACS Diva software version 8.0 (Becton, Dickinson and Company).

### Caspase 3/7 activity assay

Measurements of caspase activities in cells were performed using the commercially available Caspase-Glo 3/7 Assay (Promega, Madison, WI). Briefly, RD (1 × 104 cells/well) and RH30 (7.5 × 103 cells/well) were cultured in black 96-well plates. After 24 h of culture to allow cell adhesion, cells were incubated in the presence of zerumbone for 24 h at 37 °C in a humidified atmosphere of 5% CO_2_ in air. Caspase-3/7 activity was determined according to the manufacturer’s instructions.

### FACS analysis for quantification of apoptosis

For FACS-based apoptosis measurements RD and RH30 cells were seeded in 6-well plates at a density of 1 × 105 cells/well. Following attachment, the cells were treated in the presence of zerumbone for 72 h. After incubation the cells were collected and resuspended in 1 × Annexin—V binding buffer. To analyze the cells, 3.5 µl Annexin-V-FITC and 3.5 µl propidium iodid (PI) (50 µgml^−1^) were added to 100 µl cell suspension and incubated at room temperature for 30 min in the dark. Thereafter 200 µl Annexin—V binding buffer was added and apoptosis was analyzed using a BD FACS Canto II flow cytometer and evaluated with BD FACS Diva software version 8.0 (Becton, Dickinson and Company).

### Real-time polymerase chain reaction (RT-PCR)

Determination of NF-κB, cyclin D1, c-Myc, and CXCR4 transcript levels was performed by RT-PCR. Total RNA was extracted from RD and RH30 cells using RNeasy Mini kit (Qiagen, Hilden, Germany) according to the manufacturer’s instructions. Reverse transcription of total RNA was performed using High capacity cDNA Reverse Transcription Kit (Applied Biosystems, Waltham, Massachusetts, USA). Polymerase chain reaction (PCR) amplification of the respective genes was set up in a total volume of 20 μl using 40 ng of cDNA, 500 nM forward a reverse primer and 2 × GoTaq® qPCR Master Mix (Promega Corporation, Madison, WI, USA) according to the manufacturer’s protocol. Cycling conditions were as follows: initial denaturation at 95 °C for 5 min, followed by 40 cycles of 58 °C for 30 s and 72 °C for 20 s. The primers used for the amplification (5′–3′orientaion) are:

NFκB, fw 5′ CGA GAC AGT GAC AGT GTC TGC 3′ and rev 5′ GCT CTC TGA GCA CCT TTG GATG 3′.

Cyclin D1, fw 5′ CCG TCC ATG CGG AAG ATC–3′ and rev 5′ ATG GCC AGC GGG AAG AC–3′.

C-myc, fw 5′–AAT GAA AAG GCC CCC AAG GTA GTT ATC C–3′ and rev 5′ GTC GTT TCC GCA ACA AGT CCT CTT C–3′.

CXCR4, fw 5′ GGT TCC TTC ATG GAG TCA TAG TC 3′ and rev 5′ CGG TTA CCA TGG AGG GGA TC 3′.

TBP, fw 5′ GCC CGA AAC GCC GAA TAT 3' and rev 5' CCG TGG TTC GTG GCT CTC 3′.

Specificity of PCR product was confirmed by analysis of a melting curve. Real-time PCR amplifications were performed on a CFX96 Real-Time System (Bio-Rad). All experiments were done in duplicate. Amplification of the house-keeping gene TBP (TATA binding protein) was performed to standardize the amount of sample RNA. Relative quantification of gene expression was achieved using the ΔCt method.

### Western blotting

To analyze the expression levels of CyclinD1 and NFκB, whole cell extracts were prepared and western blot analysis was performed as originally described (Schmid et al. 2014). Antibodies directed against Cyclin D1 (1:100; Cell Signaling Technology, Inc., # 2922S) and NFκB (1:1,000; Santa Cruz, #sc109) were used as well as β-actin antibody (1∶250; Cell Signaling Technology, Inc., # 8457S) as a loading control.

### Statistics

Data are provided as means ± SEM, n represents the number of independent experiments. All data were tested for significance using unpaired Student *t* test (Welch correction) or ANOVA (Dunnett correction). Only results with *p* < 0.05 were considered statistically significant.

## Results

### The influence of zerumbone on viability of RMS cells

**Fig. 1:**
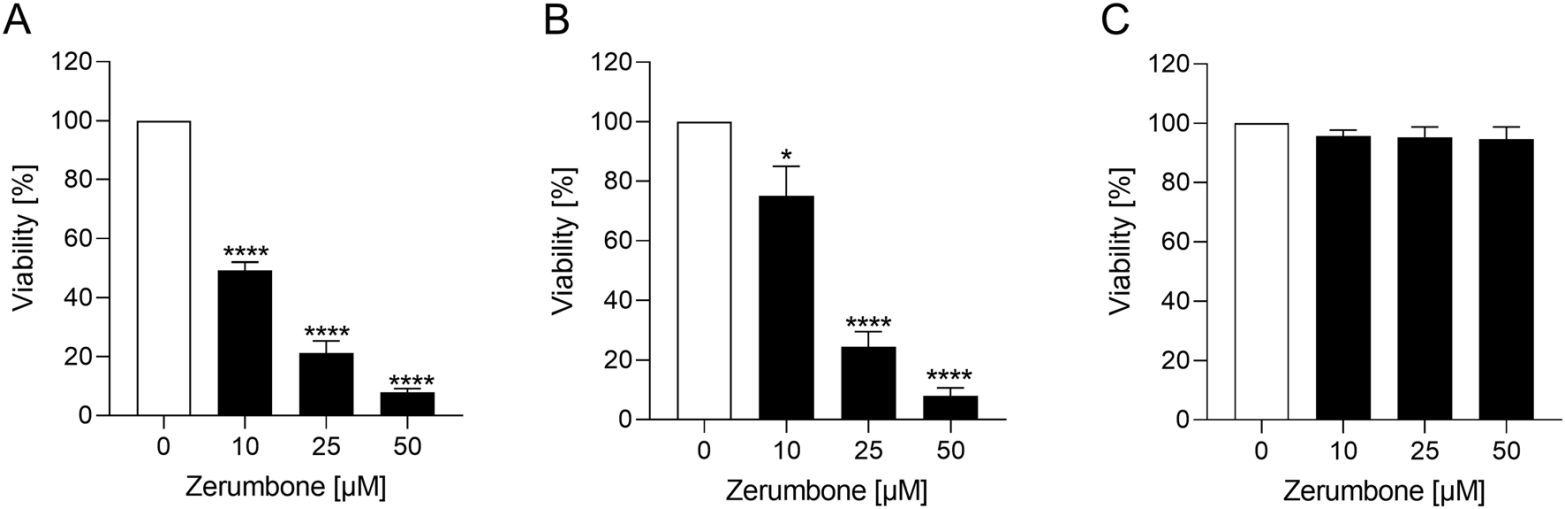
The effect of zerumbone on the viability of RD and RH30 cells as well as SkMCs. Arithmetic means ± SEM (*n* = 4) of the relative numbers of viable RD (**a**), RH30 (**b**), and SkMCs (**c**) after 72 h incubation in the presence of zerumbone (*black bars*) relative to the untreated control (*white bar*). *(*p* < 0.05), ****(*p* < 0.0001), indicate statistical significance to untreated control (ANOVA, Dunnett correction)

The cytotoxic effect of zerumbone on RMS cells was assessed. The response to different doses of zerumbone is illustrated in the Fig. 1 as percentage value of viable cells compared to untreated control sample (set at 100%). Treatment with zerumbone resulted in a significant dose-dependent decline of cell viability in both RD and RH30 cells. In SKMC cells, there was no decrease in viability even at a high concentration of 50 µM zerumbone (Fig. 1c).

### The influence of zerumbone on spheroid forming capacity of RMS cells

**Fig. 2:**
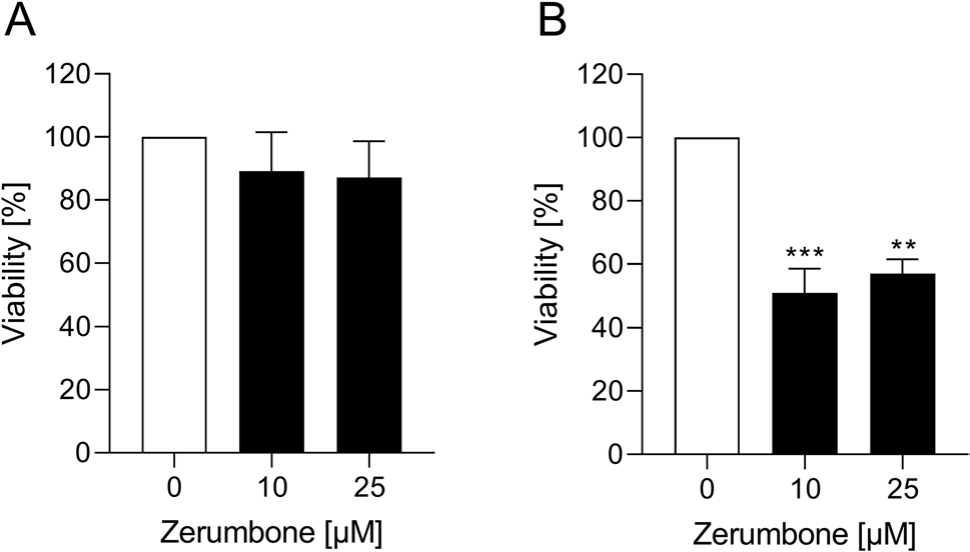
The influence of zerumbone on viability of spheroids. Arithmetic means ± SEM (*n* = 4) of the relative numbers of viable RD (**a**) and RH30 spheroids (**b**) following 72 h incubation in the presence of zerumbone (*black bar*) relative to the absence of zerumbone (*white bar*). **(*p *< 0.01); ***(*p* < 0.001) indicates statistical significance to untreated control (ANOVA, Dunnett correction)

In RH30 cells, the treatment with zerumbone resulted in a significant decrease in the viability of treated cell spheroids compared with the control, as depicted in Fig. 2b. In RD cells, no effect on spheroid viability was detected (Fig. 2a).

### Migratory response of RMS cells to zerumbone

**Fig. 3:**
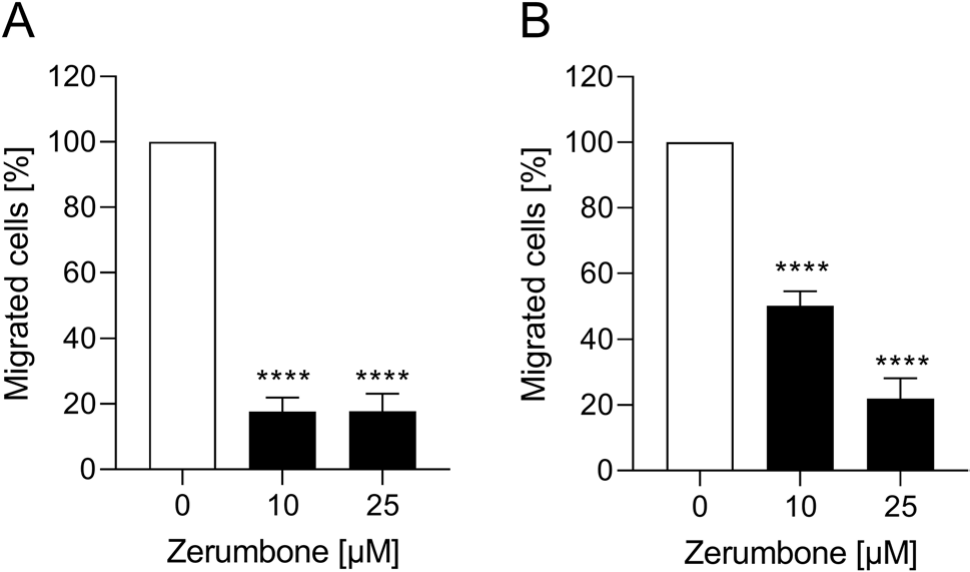
The effect of zerumbone on migration of RMS cells. Arithmetic means ± SEM (*n* = 5) of the percentage of migrated RD (**a**) and RH30 cells (**b**) in the absence (*white bar*) and presence of 10 µM and 25 µM zerumbone (*black bars*). ****(*p* < 0.0001) indicates statistically significant difference to untreated control (ANOVA, Dunnett correction)

An additional series of experiments tested the functional impact of zerumbone on cell migration of RMS cells. As shown in Fig. 3, the treatment of RD and RH30 cells with zerumbone significantly inhibited cell motility. In RD cells, reduced migration by about 80% could be observed even at a small treatment concentration (10 µM). On the other hand, the exposure of RH30 cells to zerumbone resulted in a significantly decrease of migration in a dose-dependent manner: the treatment with 10 µM zerumbone resulted in a decrease of migration by 50%, while 25 µM zerumbone determined the reduction of cell mobility by about 80%.

### The influence of zerumbone on colony-forming capacity of RMS cells

**Fig. 4:**
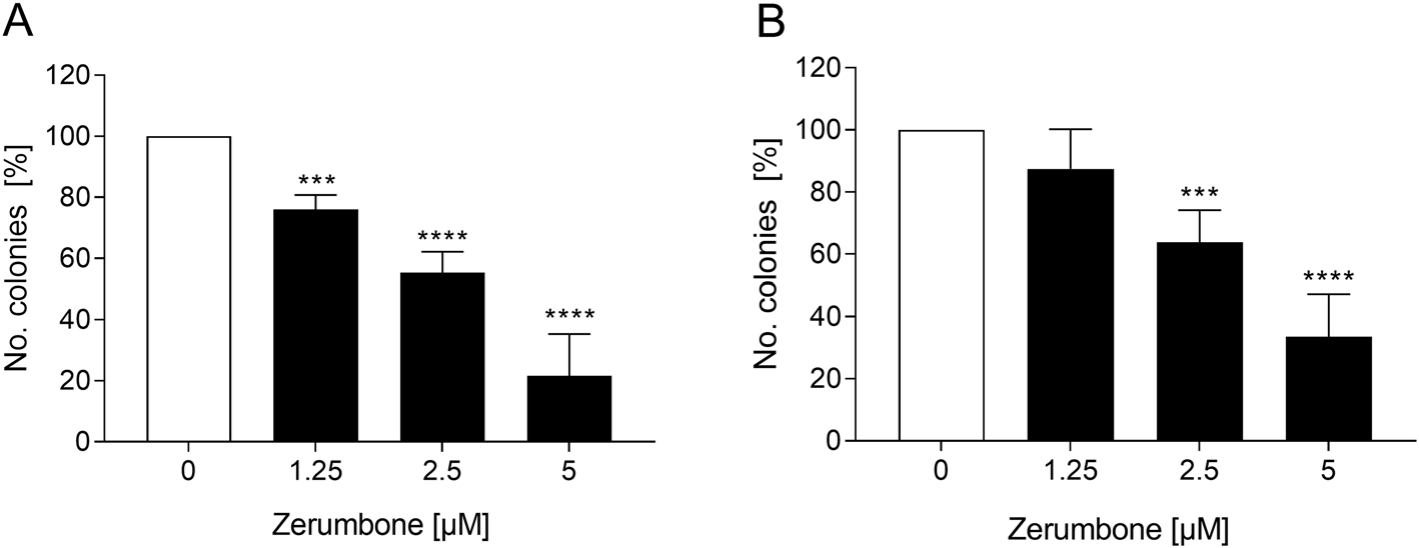
The effect of zerumbone on colony-forming capacity of RMS cells. Arithmetic means ± SEM (*n* = 5) of the relative numbers of evolving clones of RD (**a**) and RH30 (**b**) cells following incubation for 72 h in the absence (*white bars*) and presence of various concentrations of zerumbone (*black bars*). ***(*p* < 0.001), ****(*p* < 0.0001) indicate statistically significant difference to untreated control (ANOVA, Dunnett correction)

To investigate whether zerumbone exerts an effect on colony-forming capacity of RMS cells, a colony-forming assay was performed. As shown in Fig. 4, the relative numbers of colonies of RMS cells was significantly decreased in a dose-dependent manner. For example, in the presence of 5 µM zerumbone, the reduction of migration was about 85% in RD cells and 65% in RH30 cells, respectively.

### Effects of zerumbone on production of reactive oxygen species (ROS), on caspase 3/7 activity, and induction of apoptosis in RMS cells

**Fig. 5:**
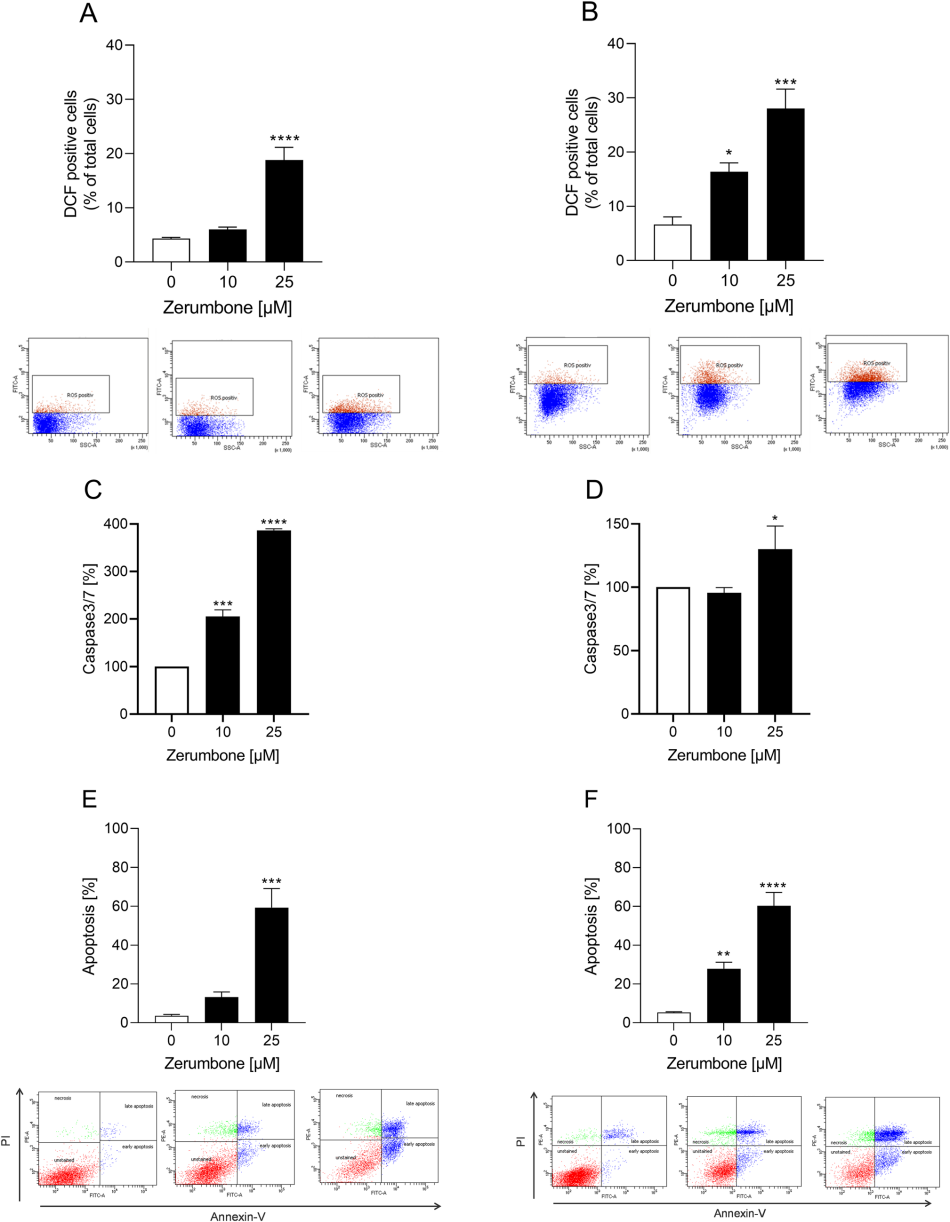
The effect of zerumbone production of reactive oxygen species (ROS), Caspase 3/7 activity, and on induction of apoptosis on RMS cells. ROS level measurement after treatment with two different concentrations of zerumbone in RD (**a**) and RH30 cells (**b**) for 24 h. Cells were treated with DCFDA and measured via flow cytometry. Caspase 3/7 activity was measured in RD (**c**) and RH30 cells (**d**) after 24 h treatment with zerumbone; Data are shown as arithmetic means ± SEM (*n* = 5). *(*p* < 0.05), ***(*p* < 0.001), ****(*p* < 0.0001) indicate statistical significance (ANOVA, Dunnett correction). Arithmetic means ± SEM (*n *= 4) of the relative numbers of viable RD (**e**) and RH30 (**f**) cells after incubation with zerumbone for 72 h incubation with 10 µM and 25 µM zerumbone (*black bars*) relative to the absence of the treatment (*white bar*). **(*p* < 0.01), ***(*p *< 0.001), ****(*p* < 0.0001) indicates the statistical significance (ANOVA, Dunnett correction)

To investigate whether the significant reduction in cell viability was due to upregulation of ROS production, increase in caspase 3/7 activity and subsequent induction of apoptosis, RD and RH30 cells were incubated with zerumbone and various analyses were performed. ROS production increased dose-dependently and significantly in both RMS cell lines (Fig. 5a, b). Incubation with 25 µM zerumbone increased ROS production by almost 20% in both cell lines.

It was further seen that caspase 3/7 activity also increased significantly in RD as well as RH30 cells (Fig. 5c, d). In RD cells, the value reached around 400% when incubated with 25 µM zerumbone. However, even 10 µM caused an increase in caspase 3/7 activity to 200%. In RH30 cells, a significant increase was only seen at 25 µM. Furthermore, the proportion of apoptotic cells, in both cell lines was significantly higher after exposure to zerumbone compared to control, as shown in Fig. 5e, f. In both cell lines, the highest level of apoptosis (60%) was reached by a zerumbone concentration of 25 µM.

### Zerumbone down-regulates the expression of nuclear factor-κB (NF-κB) and its related target genes/gene products

**Fig. 6:**
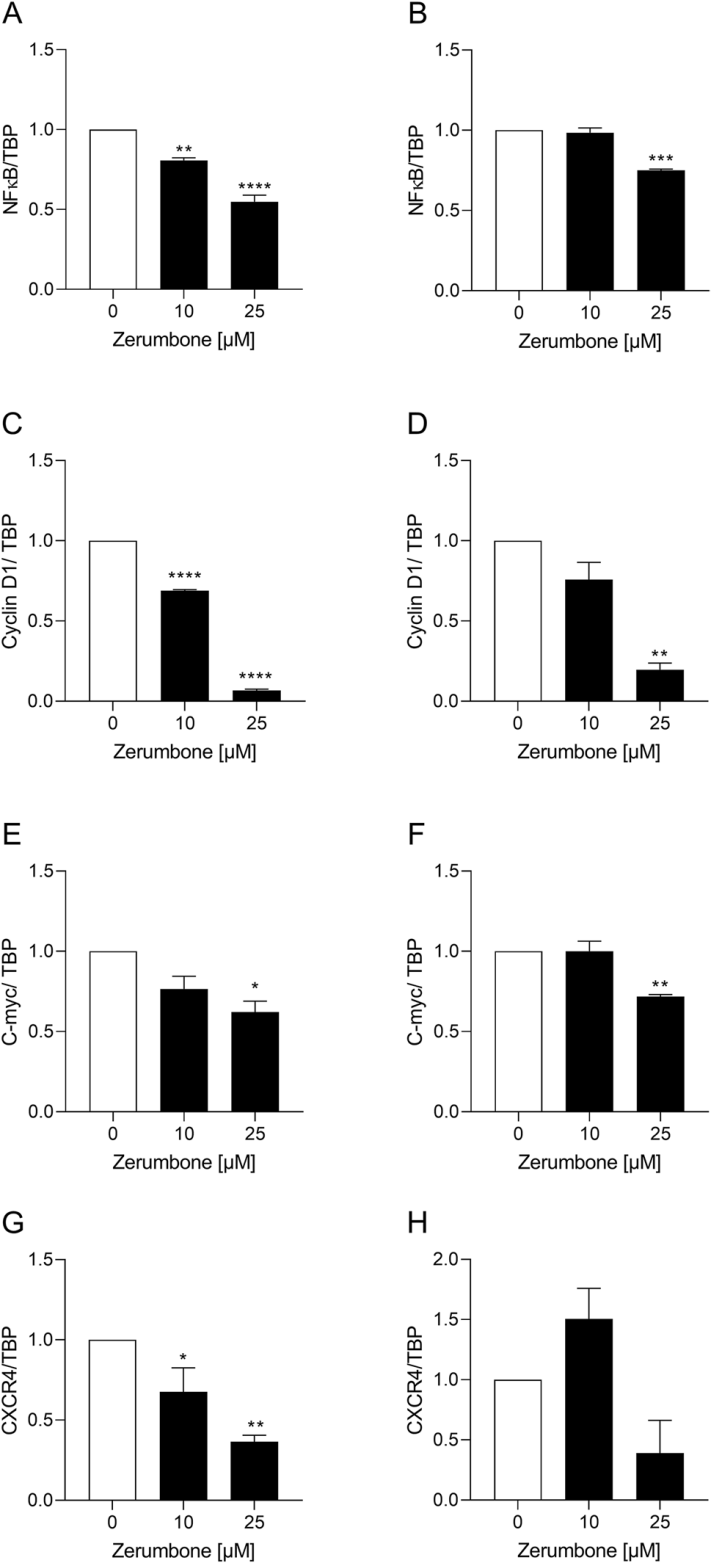
The influence of zerumbone on c-Myc expression in RMS cells. Transcriptional expression of NF-κB, cyclin D1, C-myc, and CXCR4 in RD (**a, c, e, g**) and RH30 cells (**b, d, f, h**). For quantification of mRNA levels, quantitative RT-PCR was performed using TBP as internal control. The expressions are given as relative values against to untreated control. *(*p* < 0.05), **(*p* < 0.01), ***(*p* < 0.001), ****(*p* < 0.0001) indicate statistical significance (ANOVA, Dunnett correction); (*n* = 3)

The NF-κB comprises a family of transcription factors involved in the regulation of a wide variety of biological responses. Growing evidences support a major role of this factor in oncogenesis (Dolcet et al. 2005) as NF-κB regulates the expression of genes involved in many processes that play a key role in the development and progression of cancer such as proliferation, migration, and apoptosis (Dolcet et al. 2005). Activation of NF-κB has been detected in many human malignancies (Dolcet et al. 2005). Zerumbone has been demonstrated to downregulate the NF-κB-regulated gene products including cyclin D1 and c-Myc (Rahman et al. 2014). Additionally, it was shown that zerumbone inhibits the CXCR4 expression suggesting the potential of the compound in the suppression of metastasis (Rahman et al. 2014). Therefore, we explored whether zerumbone has an influence on NF-κB, cyclin D1, c-Myc, and CXCR4 expression in RMS cells. We observed that the incubation of RD and RH30 cells with zerumbone significantly decreased the expression of NF-κB, cyclin D1, c-Myc, and CXCR4 in RD (Fig. 6a, c, e, g) and in RH30 (Fig. 6b, d, f, h) cells in a dose-dependent manner as illustrated in Fig. 6a–e.

### Modulation of NF-κB and CyclinD1 after treatment with zerumbone

**Fig. 7:**
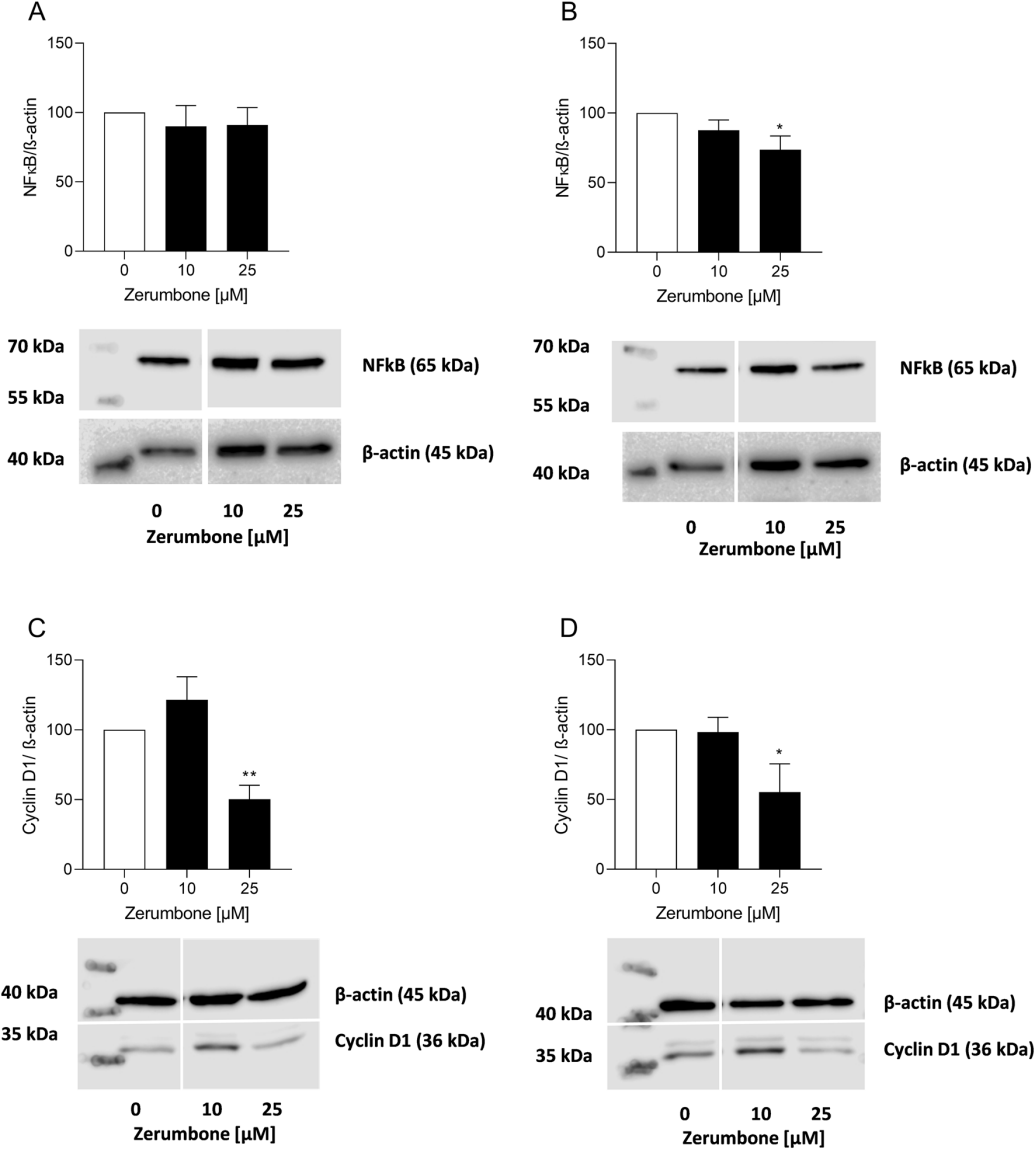
The influence of zerumbone on expression and abundance levels of NF-κB and cyclin D1. Arithmetic means (*n* = 3) of protein levels of NF-κB and cyclin D1 in RD (**a**, **c**) and RH30 cells (**b**, **d**) before (*white bars*) and after treatment with Zerumbone (*black bars*); *(*p* < 0.05) and **(*p* < 0.01) indicate statistically significant difference compared to control (ANOVA, Dunnett correction); β-actin was used as loading control

The present study further investigated the protein levels of NF-κB and Cyclin D1 in the RMS cell lines RD and RH30. Western blot experiments revealed a significant lower NF-κB and CyclinD1 abundance in RD and RH30 cell line when incubated with 25 µM zerumbone (Fig. 7b–d). Only in RD cells non-significant decrease in NF-κB abundance was observed (Fig. 7a).

## Discussion

In the present study, we investigated the anticancer effects of zerumbone on RMS cells. It was demonstrated that exposure of RD and RH30 cells to zerumbone resulted in growth inhibition, decreased proliferation, and induction of apoptosis. To our knowledge this is the first report in the literature addressing the influence of zerumbone on RMS cells.

Zerumbone is a natural dietary compound which is known to have anti-inflammatory, anti-oxidant, anti-hypertensive, and immunomodulatory biomedical properties (Girisa et al. 2019; Ghasemzadeh et al. 2017). Additionally, it can also function as an anti-tumor drug exerting its effects on proliferation and apoptosis against different types of cancer through the modulation of various proteins and signaling pathways (Girisa et al. 2019; Abdelwahab et al. 2012; Prasannan et al. 2012; Chan et al. 2015; Sun et al. 2013; Eguchi et al. 2007; Shamoto et al. 2014; Sung et al. 2008). However, the effect of zerumbone on RMS cells remains unknown.

We observed that zerumbone exerts a cytotoxic effect on RMS cells. The incubation of RD and RH30 cells with zerumbone resulted in a dramatically decrease of cell viability in a dose-dependent manner. The highest effect was encountered at a concentration of 50 µM. At this concentration, a decrease of cell viability by more than 90% could be observed in both cell lines compared with SkMCs. These findings are consistent with the existing literature. Zhang et al. has reported that incubation of pancreatic cancer cells (PANC-1) with zerumbone resulted in a decrease of cell viability in a dose and time-dependent manner (Zhang et al. 2012). The highest reduction of cell viability (50%) was reached at a concentration of 100 µM. It is important to mention again that zerumbone did not affect the viability of SKMC cells, even at a high concentration of 50 µM.

There are many reports which demonstrate that zerumbone determines induction of apoptosis in cancer cells. The mechanisms through which zerumbone induce apoptosis depends on cancer cell type. In liver cancer cells, the apoptotic process triggered by zerumbone has been reported to involve the upregulation of pro-apoptotic Bax protein and the suppression of anti-apoptotic Bcl-2 protein expression (Sakinah et al. 2007). In esophageal cancer, zerumbone-triggered downregulation of Bcl-2 expression has also been proven to be responsible for induction of apoptosis (Ma et al. 2018). On the other side, Yodkeeree et al. reported that zerumbone had no effect on Bcl-2 family proteins in human colon cancer cells (Yodkeeree et al. 2009). In non-small cell lung cancer zerumbone has been demonstrated to induce apoptosis through loss of mitochondrial membrane potential, release of cytochrome c, caspase-9 and caspase-3 activation and increased ROS production (Hu et al. 2014). Apoptosis through caspase-3 activation has also been demonstrated after treatment with zerumbone in renal cell carcinoma cells and pancreatic cancer cells (Sun et al. 2013; Zhang et al. 2012).

Generation of ROS represent one of the main biochemical changes central to the mechanism of apoptosis. It has previously been reported that zerumbone can potentiate TRAIL-induced apoptosis through generation of ROS in human colon cancer cells (Yodkeeree et al. 2009). Increased ROS production after zerumbone treatment has also been reported in chronic myelogenous leukemia cells, and pancreatic cancer cells (Zhang et al. 2012; Rajan et al. 2015). In line with these results, we also observed that the induction of apoptosis is through a substantial increase in ROS generation in RMS cells in a dose-dependent manner and the upregulation of the caspase 3/7 activity.

Zerumbone possesses antiproliferative properties toward several cancer cell lines with minimal effect on normal cells (Rahman et al. 2014; Prasannan et al. 2012). For example, zerumbone was reported to inhibit the proliferation of leukemia cells by induction of G2/M cell cycle arrest and mithocondria-mediated apoptosis (Xian et al. 2007). Zerumbone has also been found to decrease the proliferation of human breast cancer cells and that the growth inhibitory effect of zerumbone was associated with cell cycle arrest and apoptosis induction (Sehrawat et al. 2012). Additionally, zerumbone has been shown to strongly inhibit the proliferation of liver cancer cells and enhance the apoptosis (Sakinah et al. 2007). Our results are in conformity with these findings. We observed that zerumbone, even in smaller concentrations dramatically decreased the number of colonies of RD and RH30 cells by about 85% and 65% respectively. Additionally, the treatment of both cell lines with zerumbone significantly inhibited the cell motility.

Zerumbone exerts its anti-inflammatory, antiproliferative, and antimigratory properties also through the modulation of NF-κB activity (Rahman et al. 2014). This compound also down-regulates NF-κB-regulated gene products including cyclin D1 and c-Myc. Cyclin D1 is an important regulator of cell cycle progression and is required for progression through G1 phase of the cell cycle to induce cell migration (Neumeister et al. 2003). Mutations, amplifications and overexpression of this gene, which alters cell cycle progression are observed frequently in a variety of tumors and may contribute to tumorigenesis (Neumeister et al. 2003). The cellular homolog (c-Myc) of the viral gene v-Myc controls the transition from G1 to S phase. It has been reported that inhibition of c-Myc expression determines growth arrest (Gupta et al. 2010). In our study we observed that the expression of NF-κB, cyclin D1, and c-Myc was significantly down-regulated in both cell lines after exposure to zerumbone. In RH30 cells, a significantly reduced expression of cyclin D1-mRNA could be observed only at a concentration of 25 µM. This may suggest the fact that in RMS cells zerumbone exerts its antiproliferative and antimigratory effects through regulation of NF-κB pathway and its regulated gene products.

It has been reported that zerumbone is a novel inhibitor of chemokine receptor-4 (CXCR4) expression, which mediates homing of tumor cells to specific organs during metastasis, suggesting the potential of the product in the suppression of cell metastasis (Rahman et al. 2014; Sung et al. 2008). Sun et al. reported that downregulation of NF-κB by zerumbone mediated downregulation of CXCR4 in breast cancer cells (Sun et al. 2013). The CXCR4-SDF1α axis has been reported to be involved in the migration and metastatic invasion of RMS cells in vitro (Regenbogen et al. 2020). Our group has previously reported that the expression levels of CXCR4 significantly increased the migratory behavior of RH30 cells. Additionally, treatment of RH30 cells with an CXCR4 antagonist in combination with doxorubicin and vincristine determined a dramatic reduction of RH30 cells migration (Regenbogen et al. 2020). In the present study we found that the treatment of RMS cells with zerumbone resulted in a significant decreased expression of CXCR4 in both cells lines, suggesting that this compound may arrest the cell migration through downregulation of CXCR4 expression.

In conclusion, the present study confirms that zerumbone is a promising candidate for the anticancer therapy due to strong inhibitory and apoptotic effects on pediatric rhabdomyosarcoma cell lines through increased caspase-3/7 activity and increased ROS production as well as through the modulation of NF-κB pathway and merit further investigation including in vivo analysis.
